# Elucidation of E3 ubiquitin ligase specificity through proteome-wide internal degron mapping

**DOI:** 10.1016/j.molcel.2023.10.021

**Published:** 2023-11-16

**Authors:** Zhiqian Zhang, Brandon Sie, Aiquan Chang, Yumei Leng, Christopher Nardone, Richard T. Timms, Stephen J. Elledge

After publication, we noticed a minor error in Figure 2A. The rows of the heatmap were out of order and did not match the peptide name labels on the left side of the graphic. This was due to a mistake made during reformatting the figure to cluster the maps corresponding to the CRISPR data below it. The corrected version of the figure now appears with the paper online. Because the purpose of Figure 2A is to show examples of peptides with similar hydrophobic degron motifs, this error does not affect the interpretation of the paper.

## Figures and Tables

**Figure 2A.**
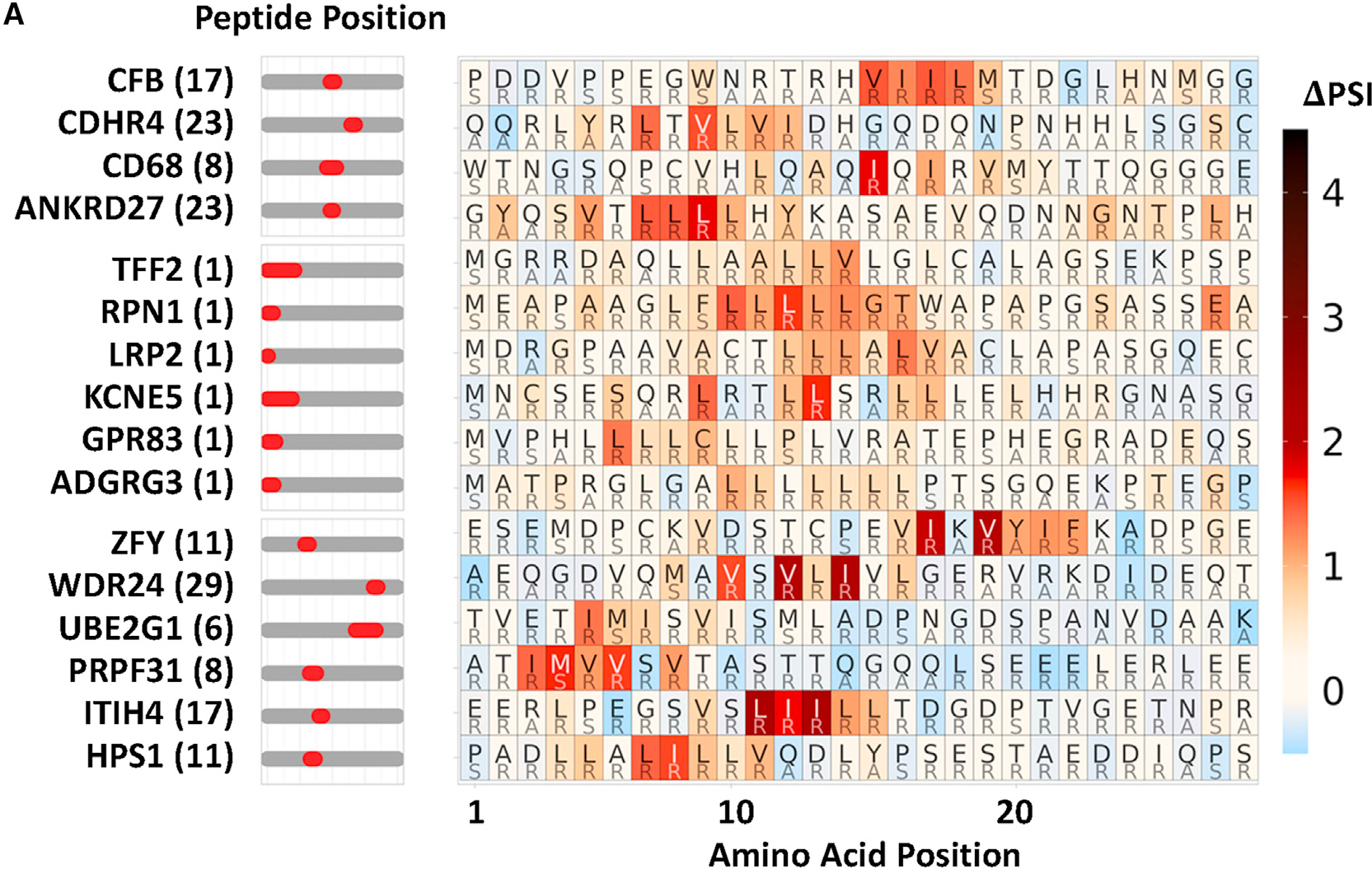
SVM machine learning-aided identification of a BAG6 degron motif (corrected)

**Figure 2A.**
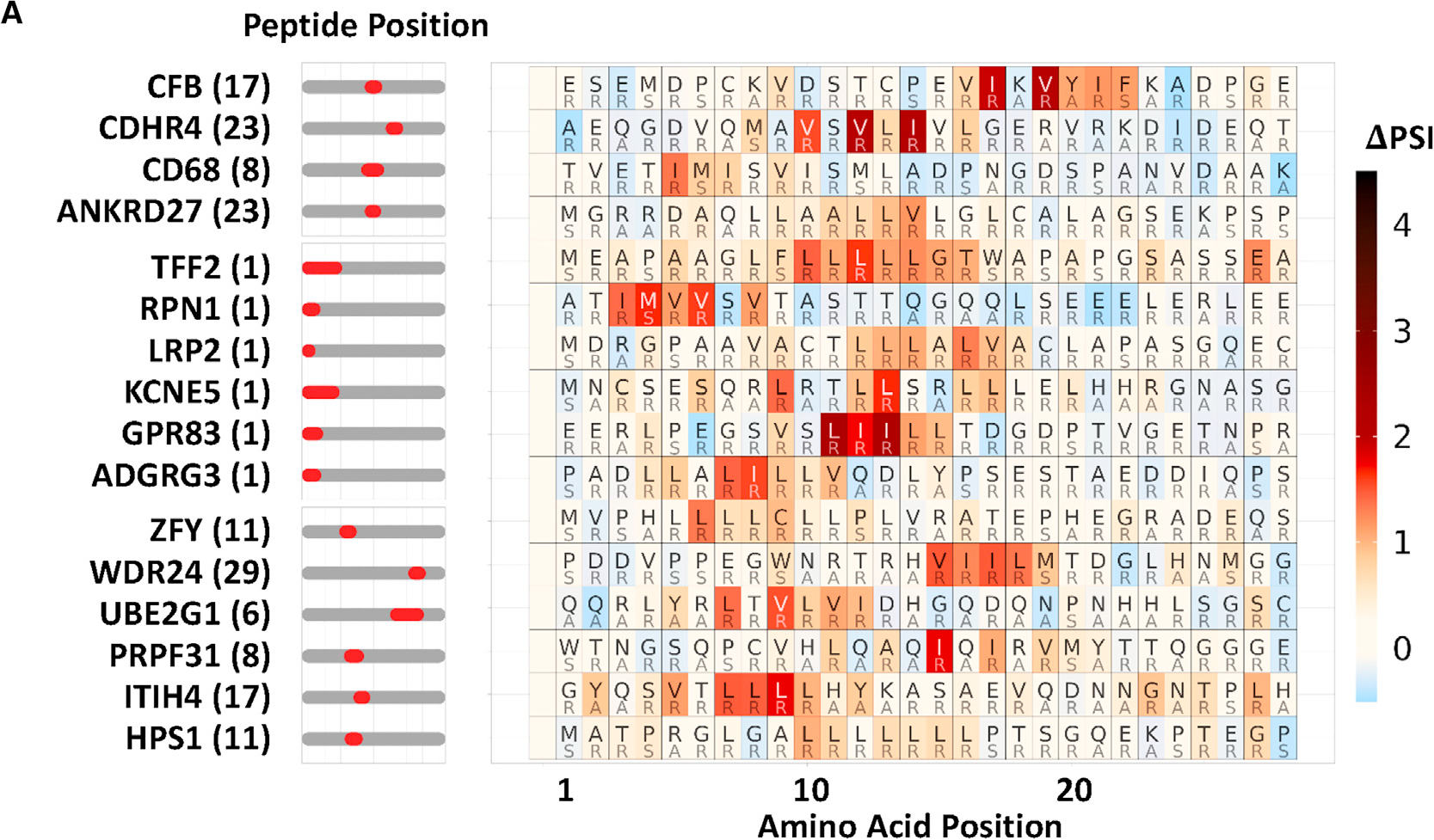
SVM machine learning-aided identification of a BAG6 degron motif (original)

